# Phosphate system fertilization in no‐tillage Oxisols: Effects of temporal, horizontal, and vertical distribution of available phosphorus in the soil

**DOI:** 10.1002/jeq2.70108

**Published:** 2025-10-24

**Authors:** Andria Paula Lima, Sandra M. V. Fontoura, Dayana Jéssica Eckert, Amanda Posselt Martins, Renan Costa Beber Vieira, Cimélio Bayer, Tales Tiecher

**Affiliations:** ^1^ Department of Soil Science Federal University of Rio Grande do Sul (UFRGS) Porto Alegre Rio Grande do Sul Brazil; ^2^ Fundação Agrária de Pesquisa Agropecuária Guarapuava Paraná Brazil; ^3^ Department of Agronomy Federal University of Fronteira Sul–Campus Cerro Largo (UFFS) Cerro Largo Rio Grande do Sul Brazil

## Abstract

System fertilization enhances logistics and phosphorus (P) use efficiency, but its effects in high P‐export systems, particularly regarding fertilization timing, placement, and distribution, remain unclear. This study evaluated P fertilization timing (90, 45, and 0 days before winter crop sowing [DBS]), placement (banded vs. broadcast), and spatial distribution (17 × 5 vs. 40 × 10 cm) in subtropical Oxisols with medium and high soil‐test P. Over 4 years, we assessed crop yields, partial phosphorus balance (PPB), and Mehlich‐1 available soil P (0–10 and 10–20 cm) under rotations of corn (*Zea mays* L.), soybean (*Glycine max* L.), and winter crops including black oat (*Avena strigosa* Schreb.), barley (*Hordeum vulgare* L.), vetch (*Vicia sativa* L.), and fodder radish (*Raphanus sativus* L.). Despite pronounced P stratification, yields and PPB were generally unaffected. In medium P soil, the 40 × 10 cm spacing increased P in the 10–20 cm layer to 61% of the critical value after 4 years. In high P soil, P application at winter sowing raised subsurface P to 4.5 mg dm^−3^. Soybean yield (5.2 Mg ha^−1^) and PPB (73%) peaked with 90 DBS banded fertilization. Anticipating fertilization by 90 days with 17 × 5 cm spacing improved soybean yield by 20% and PPB by 10% due to better surface P distribution. The 0–10 cm layer remained P‐rich and sufficient for grain yield. However, the benefits of 90 DBS were limited to two seasons. Long‐term studies are needed to refine system fertilization strategies in high‐output grain systems.

## Core Ideas

- Phosphorus fertilization time and placement minimally affect yields but alter soil P distribution in Oxisols.
- Anticipated P fertilization at 17 × 5 cm row spacing boosts soybean yield and P balance.
- Surface soil layers remain P‐rich and sufficient for crop yield despite P management variations.
- Long‐term studies are critical for optimizing P management in high‐export cropping systems.

## Plain Language Summary

Plants need phosphorus (P) to grow. Farmers use fertilizer to add P to the soil. In system fertilization, all the P is added during the winter. This study tested different ways and times to do that to see what works best for crops. The researchers wanted to find out the best way to apply P fertilizer in soils that already have medium or high levels of P. They checked how it affected crop production and how much P stayed in the soil. Most of the time, changing how and when the fertilizer was added did not make much difference because the soil already had enough P for plants. But sometimes, adding P 90 days before planting the winter crop and placing it in rows helped soybeans grow better. This way also made better use of the fertilizer. If farmers know the best way to use fertilizer, they can grow more food, spend less money, and take better care of the environment. But we still need more studies to see what works best over many years.

**Abbreviations**
CSTV: critical soil test value
DBS: days before winter crop sowing
PPB: partial phosphorus balance

## 1 INTRODUCTION

Phosphorus (P) is one of the most limiting nutrients for global agricultural production (Oliveira et al., 2022; Weil & Brady, 2017). This is especially evident in tropical and subtropical agricultural systems, where soils exhibit high Fe and Al oxide levels and have a high phosphate adsorption capacity, reducing P fertilizer use efficiency (Fink et al., 2016). To ensure the maximum productive potential of high‐demand crops such as soybeans, corn, and winter cereals, applying phosphate fertilizers is essential to meet their P requirements (Hopkins & Hansen, 2019).

In subtropical agricultural systems characterized by soils with high P adsorption capacity and intensive grain production (i.e., two harvests per year), strategies are needed to enhance nutrient use efficiency and improve operational efficiency. One emerging strategy on soils with medium and optimum nutrient availability is system fertilization, defined as a single annual application of P fertilizer, usually during winter crop sowing, to supply both winter and subsequent summer crops. This contrasts with the traditional crop fertilization, in which P is applied at the sowing of both crops. System fertilization targets winter crops since cereals are generally more nutrient‐demanding (Vieira et al., 2013, 2015), and the sowing window is broader than that of summer crops. Moreover, winter sowing allows better horizontal fertilizer distribution due to narrower row spacing (17 cm). Omitting P application in summer improves sowing logistics, which are often constrained by climatic conditions (e.g., excessive or insufficient rainfall) (Assmann et al., 2017).

System fertilization is effective in Southern Brazil's crop–livestock systems, where summer grains alternate with winter forage. Anticipating P and K application in winter boosts forage yield without reducing summer soybean yield in the initial years (Alves et al., 2022; Farias et al., 2020), as most nutrients consumed by animals return to the soil through manure and urine (Alves et al., 2022, 2019). Only about 5% of P and K are removed through animal production (via meat) in winter, with the remaining 95% cycling in the soil system (Alves et al., 2019). Over time, soybean yields may even increase with annual fertilization before grazing (Camargo et al., 2024; Simões et al., 2023) due to improved soil microbial activity (Camargo et al., 2024; Pires et al., 2022) and soil physical quality (Simões et al., 2023). However, these benefits remain unproven in grain‐only systems, especially under high productivity, high P export, and varying soil P levels.

Unexplored aspects include the temporal and spatial (horizontal/vertical) P distribution and placement methods. Anticipation of P fertilization within system fertilization could improve logistics during winter crop sowing but can increase P adsorption and reduce availability due to prolonged soil contact (Barrow, 1974; 1983). Vertically, deep P application has reduced soil P gradients and improved root access (Fernández & Schaefer, 2012; Kang et al., 2014; Yuan et al., 2020). While banded fertilization studies have addressed depth, horizontal distribution is less explored. Lu et al. (2019) found that using a 12 cm spacing between P application rows and sowing rows decreased P uptake and wheat productivity compared to other spacings.

In turn, the placement method directly influences soil P distribution and plant uptake (Oliveira et al., 2022). Due to its low mobility, P accumulates where applied (Nunes et al., 2020), especially in no‐till systems, with surface fertilizer placement (Calegari et al., 2013). In Brazil, banded and broadcast are the main fertilizer application methods (Nunes et al., 2020). While broadcast P improves operational efficiency, it tends to create strong P gradients in the soil profile (Oliveira et al., 2022; Tiecher et al., 2023). This P accumulation in the surface layers can have agronomic and environmental implications. Agronomically, it is unfavorable because the surface layers dry out first during drought periods, significantly reducing nutrient uptake by diffusion—the primary mechanism of P supply to plants (Bellinaso et al., 2021). Additionally, P accumulation at the surface exacerbates nutrient losses through surface runoff, causing economic losses and potentially significant environmental impacts (Reichert et al., 2019).

Compared to broadcast, banded P increases P uptake and grain P concentration (Hansel et al., 2017) and improves soybean, corn, and wheat yields by an average of 3.7%, reaching up to 5%–27% with nitrogen (Nkebiwe et al., 2016). It reduces soil contact, concentrating P in a smaller volume, increasing availability, and reducing vertical gradients (Nunes et al., 2011). Banded P also increases P levels in deeper soil layers (e.g., 10–20 cm) and enhances yields (Bellinaso et al., 2021). However, it may increase horizontal P variability, requiring careful soil sampling to avoid overestimations (Tiecher et al., 2023).

Beyond these factors, P fertilization management is influenced by fertilization history and soil P availability (Lopes et al., 2021). Addressing the effects of these factors under different soil‐test P conditions (medium and high) is essential, as they reflect recurring scenarios in agricultural production systems. Soils with a history of P application often create a P legacy that affects the availability of P and environmental risks (Doydora et al., 2020). Managing this legacy is key to optimizing fertilizer use and reducing losses. This study evaluated (i) fertilization timing (90, 45, and 0 days before sowing—DBS) and placement (broadcast vs. banded), and (ii) timing (90 and 45 DBS) and spatial distribution (17 × 5 vs. 40 × 10 cm) in a subtropical Oxisol under system fertilization with medium and high soil‐test P, assessing yields, P use efficiency, and available P. The hypotheses are that in soils with soil‐test P below the critical soil test value (CSTV): (i) banded P at winter sowing improves yield, efficiency, and available P versus broadcast and other timings; (ii) P applied closer to sowing (45 DBS) with greater depth and row spacing (40 × 10 cm) enhances outcomes versus 90 DBS and 17 × 5 cm. Conversely, in soils with soil‐test P above CSTV, fertilization time, placement method, and spatial distribution are expected to have a low impact on the evaluated parameters.

**Core Ideas**

- Phosphorus fertilization time and placement minimally affect yields but alter soil P distribution in Oxisols.
- Anticipated P fertilization at 17 × 5 cm row spacing boosts soybean yield and P balance.
- Surface soil layers remain P‐rich and sufficient for crop yield despite P management variations.
- Long‐term studies are critical for optimizing P management in high‐export cropping systems.

## 2 MATERIALS AND METHODS

### 2.1 Site description

**FIGURE 1:**
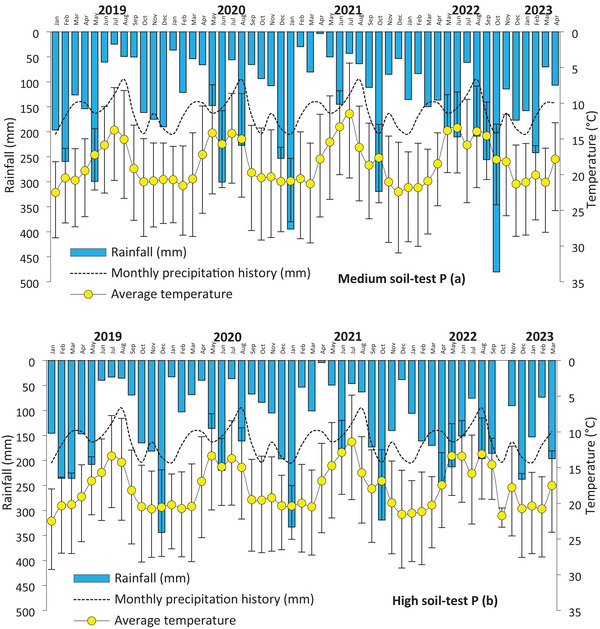
Average monthly air temperature (average with and bars that indicates maximum and minimum values), monthly precipitation history (1976–2019—IDR Paraná, 2024), monthly rainfall in the field trials with medium (a: Candói, PR) and high (b – Pinhão, PR) soil‐test P conditions during the study period (2019–2023). In the South Hemisphere, winter cereals are grown from June to October, soybean from November to March, and corn from September to February.

Two field trials were conducted in the Center‐South of Paraná State, Southern Brazil, with a humid subtropical climate (Cfb type, Köeppen). Altitudes range from 800 to 1200 m, with an average annual temperature of 17°C (21°C in summer, 13°C in winter). The average annual precipitation is 1921 mm, well distributed throughout the year with no dry season and frequent frosts (Fontoura et al., 2015). The soils in both field trials are classified as Oxisols (Humic Hapludox) (Soil Survey Staff, 2022) with clayey to very clayey texture. In the clay fraction, the predominant minerals are kaolinite, iron oxides (goethite and hematite), and aluminum oxides (gibbsite) (Inda et al., 2007). Both field trials were carried out for 4 years in areas under no‐tillage for >20 years, presenting medium (Candói site) and high (Pinhão site) soil‐test P levels. Available soil P levels extracted by Mehlich‐1 followed the recommendations of Vieira et al. (2015) for the Center‐South region of Paraná: Low *p* < 4 mg dm^−3^_,_ Medium *p* = 4–8 mg dm^−3^, High *p* = 8–16 mg dm^−3^, Very high >16 mg dm^−3^. The CSTV of P by Mehlich‐1 in the 0–20 cm soil layer in this region is 8 mg dm^−3^ (Vieira et al., 2015). Some soil characteristics of both field trials are presented in Table 1. The monthly rainfall and temperature of the field trials from 2019 to 2023 are shown in Figure 1.

**TABLE 1:** Selected soil properties of the 0–20 cm layer before the establishment of the field trials in the sites with medium soil‐test P (Candói) and high soil‐test P (Pinhão), State of Paraná, Southern Brazil.

Soil properties	Medium soil‐test P (Candói, PR)	High soil‐test P (Pinhão, PR)
Clay content^a^ (g kg^−1^)	>600	>600
Soil pH in CaCl_2_ ^b^	5.2	5.6
Total organic carbon^c^ (g kg^−1^)	38	34
Cation exchange capacity^d^ (cmol_c_ dm^−3^)	17.5	11.9
Exchangeable Al^e^ (cmol_c_ dm^−3^)	0.0	0.0
Exchangeable Ca^e^ (cmol_c_ dm^−3^)	7.2	4.6
Exchangeable Mg^e^ (cmol_c_ dm^−3^)	4.0	2.4
Available K^f^ (cmol_c_ dm^−3^)	0.2	0.4
Available P^f^ (mg dm^−3^)	4.9	15.9
Saturation by Ca + Mg + K^g^ (%)	65	62

^a^ Pipette method.

^b^ pH in CaCl_2_ (0.01 mol L^−1^), relation 1:2.5.

^c^ Walkley‐Black method (K_2_Cr_2_O_2_ 0.5 mol L^−1^).

^d^ Sum of Ca + Mg + K + (H + Al).

^e^ Extracted by KCl 1.0 mol L^−1^.

^f^ Extracted by Mehlich‐1 solution (HCl 0.05 mol L^−1^ + H_2_SO_4_ 0.0125 mol L^−1^).

^g^ (Ca + Mg + K/CEC_pH 7.0_) × 100.

### 2.2 Experimental design and treatments

**FIGURE 2:**
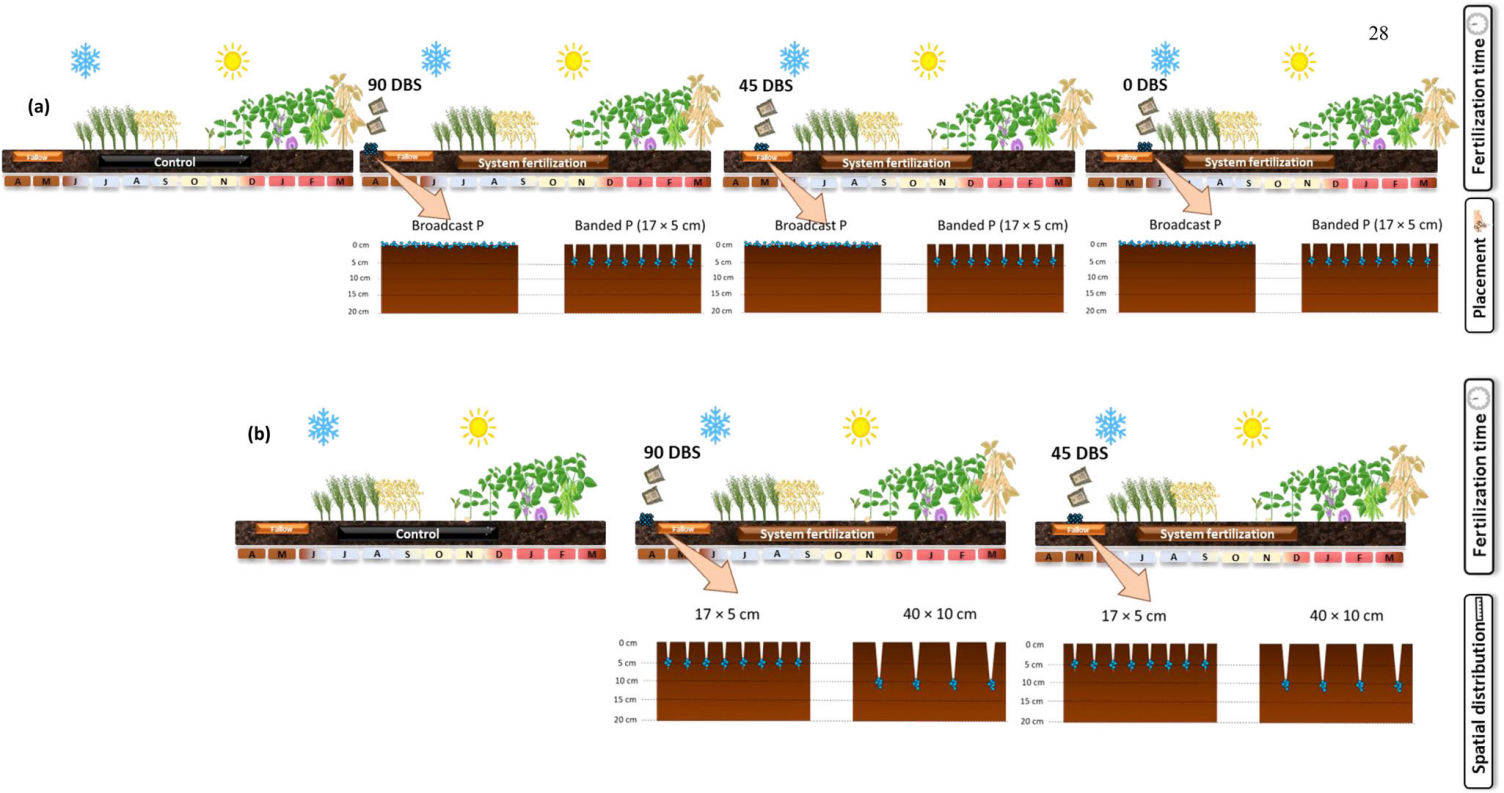
Scheme representing the two analyses for variance (ANOVA) tested in the field trials established in Oxisols with medium (Candói) and high soil‐test P (Pinhão), State of Paraná, Southern Brazil: Fertilization time × placement (ANOVA 1) (a) and fertilization time × spatial distribution (ANOVA 2) (b). DBS, days before sowing.

Field trials were established in 2019 to evaluate three experimental factors related to phosphate fertilization: (i) fertilization timing, (ii) placement method, and (iii) spatial distribution (Table 2). Timing treatments consisted of phosphorus (P) applied at 90, 45, and 0 days before winter crop sowing (DBS). The 90 and 45 DBS applications were performed in autumn over summer crop residues, and plots were maintained fallow until winter sowing (Figure 2a).

**TABLE 2:** Experimental factors (fertilization time, placement method, and spatial distribution) applied on the field trials with medium and high soil‐test P on no‐till Oxisols, State of Paraná, Brazil.

Fertilization time	Placement method	Spatial distribution	ANOVA 1	ANOVA 2
Control	Control	Control	X	X
0 DBS	Banded	–	X	–
0 DBS	Broadcast	–	X	–
45 DBS	Banded	17 × 5 cm	X	X
45 DBS	Banded	40 × 10 cm	–	X
45 DBS	Broadcast	–	X	–
90 DBS	Banded	17 × 5 cm	X	X
90 DBS	Banded	40 × 10 cm	–	X
90 DBS	Broadcast	–	X	–

*Note*: Analysis of variance (ANOVA) 1: Fertilization time (90, 45, and 0 DBS) and placement (banded P and broadcast P); ANOVA 2: Fertilization time (90 and 45 DBS) and spatial distribution (17 × 5 and 40 × 10 cm).

Abbreviation: DBS, days before sowing winter crop.

Placement method treatments included (i) banded P application at a 5‐cm depth and (ii) surface broadcast application. For the spatial distribution factor, P was applied using either: (i) 17 cm horizontal spacing at 5‐cm depth (17 × 5 cm) or (ii) 40 cm horizontal spacing at 10‐cm depth (40 × 10 cm) (Figure 2b). A no‐P control was also included. Treatments were arranged in a randomized block design with three (medium soil‐test P) or four (high soil‐test P) replicates. In the medium soil‐test P trial, plots measured 3.2 m in width by 10 m in length, resulting in a plot area of 32 m^2^. Plot dimensions were 3.2 × 10 m (32 m^2^) for medium and 3.2 × 11 m (35 m^2^) for high P sites. Winter crops were sown in rows spaced 17 cm apart (∼18 rows/plot). Summer crops such as soybean (*Glycine max* L.) and corn (*Zea mays* L.) were sown at 40 cm (eight rows/plot) and 80 cm (four rows/plot) spacing, respectively.

### 2.3 Crop rotations and phosphate fertilization

This study covered four agricultural years, with crop rotations differing between the medium and high soil‐test P sites (Table 3). In the medium P site, winter crops included *Avena strigosa* Schreb. (black oat, three seasons), *Vicia sativa* (common vetch, one season), and *Hordeum vulgare* L. (barley, one season). Summer crops included *Glycine max* L. (soybean, three seasons) and *Zea mays* L. (corn, one season). In the high P site, winter crops included *Avena strigosa* Schreb. (three seasons), *Raphanus sativus* L. (fodder radish, one season), and *Hordeum vulgare* L. (one season). Similarly, *Glycine max* L. was cultivated for three seasons and *Zea mays* L. for one season during summer (Table 3).

**TABLE 3:** Crop rotation in each experimental site and the amount of P applied according to each year in the field trials with medium and high soil‐test P condition in Oxisols under no‐tillage located in the Center‐South of Paraná.

Period	Year	Medium soil‐test P (Candói, PR)		High soil‐test P (Pinhão, PR)	
		Crop	P rate (kg ha^−1^)	Crop	P rate (kg ha^−1^)
First year	Winter 2019	Black oat	83	Black oat	50
	Summer 2019/2020	Soybean	0	Soybean	0
Second year	Winter 2020	Barley	83	Black oat	68
	Summer 2020/2021	Soybean	0	Corn	0
Third year	Winter 2021	Black oat	83	Fodder radish/Barley	68
	Summer 2021/2022	Soybean	0	Soybean	0
Fourth year	Winter 2022	Black oat/Vetch	67	Black oat	0
	Summer 2022/2023	Corn	0	Soybean	0
Total			317		186

Phosphorus rates (Table 3) were based on official recommendations for the South‐Central region of Paraná (Fontoura et al., 2015). Under the system fertilization strategy, the full recommended rate (100%) was applied during the winter crop. Across the 4 years, total P application amounted to 317 kg P ha^−1^ in the medium soil‐test P site and 186 kg P ha^−1^ in the high soil‐test P site, using triple superphosphate as the P source in all seasons (Table 3).

### 2.4 Plant evaluations and performance index

Black oat dry matter yield was assessed at the beginning of grain filling and expressed in Mg ha^−1^. Soybean, corn, and barley crops were evaluated for grain yield at 130 g kg^−1^ moisture and also expressed in Mg ha^−1^. The export of P via grains was estimated using the yield of each crop and the P concentration in the grains. For barley, the P concentration in the grain used was 3.0 kg Mg^−1^, which was obtained from a local database of the Agrária Foundation for Agricultural Research. For corn, the P concentration in the grain used was 2.2 kg Mg^−1^, which was the average concentration obtained from 36 observations from Sena (2010) and 41 from Duarte et al. (2019). For soybean, the P concentration in the grain used was 5.5 kg Mg^−1^, obtained from a meta‐analysis of soybeans in Brazil (Filippi et al., 2021). From the yield and P export data via grains, the partial phosphorus balance (PPB) was calculated as an efficiency index that expresses how much nutrient is being removed from the system when compared to how much is being applied (Dobermann, 2007) (Equation 1).

(1) $$\begin{array}{ccc} & & \mathrm{Partial}\;\mathrm{phosphorus}\;\mathrm{balance}\;\;\left(\%\right)\\ & & \;=\;\frac{P\;\mathrm{remove}\;\mathrm{by}\;\mathrm{grain}\;\left(\mathrm{kg}\;ha^{-1}\right)}{\;P\;\mathrm{applied}\;\mathrm{via}\;\mathrm{fertilizer}\;\left(\mathrm{kg}\;ha^{-1}\right)}\times 100 \end{array}$$

### 2.5 Soil sampling and analysis

Soil samples were collected after the summer crop harvests in 2019/2020, 2021/2022, and 2022/2023, corresponding to the first, third, and fourth years of experimental evaluations. In each plot, a composite sample was formed by collecting three subsamples with a shovel across the crop row, from one inter‐row to the next. Samples were taken from two soil layers: 0–10 and 10–20 cm. All samples were air‐dried, mechanically ground, and sieved through a 2‐mm mesh. Available P was extracted using the Mehlich‐1 solution (0.05 mol L^−1^ HCl + 0.0125 mol L^−1^ H_2_SO_4_) (Mehlich, 1953), following the procedures described by Tedesco et al. (1995). Available P in 0–20 cm was estimated as the average of the two layers.

### 2.6 Statistical analysis

Data were tested for normality (Shapiro–Wilk test) and homogeneity of variance (Bartlett's test). When necessary, Box‐Cox transformations were applied to variables such as available P and grain yield to meet these assumptions. Analysis of variance (ANOVA) was performed at a 0.05 significance level, and when significant, treatment means were compared using Tukey's test (*p* < 0.05). For each soil‐test P condition, the data were organized into two ANOVA analyses: (i) Fertilization time × placement method (Figure 2a) and (ii) Fertilization time × spatial distribution (Figure 2b). The statistical analyses were conducted as two bifactorial ANOVAs, as indicated in Table 2. Also, control was considered an additional treatment. The effects included in the statistical model were placement method (banded P or broadcast), fertilization time (90, 45, or 0 DBS), spatial distribution (17 × 5 or 40 × 10 cm), and soil layer (0–10 or 10–20 cm) for soil P data. These effects and their interactions were treated as fixed effects, while the block was treated as a random effect. Additionally, available P in each layer and crop yields from the control were compared with the P‐applied treatments mean using the Wilcoxon signed–rank test (*p* < 0.05; non‐parametric). The cumulative grain yield for seasons and P use efficiency indices were presented with standard error bars. All analyses were performed using RStudio version 4.2.0.

## 3 RESULTS

### 3.1 Evolution of the available P in‐depth and its effect on crop yields

#### 3.1.1 Fertilization time versus placement method

**FIGURE 3:**
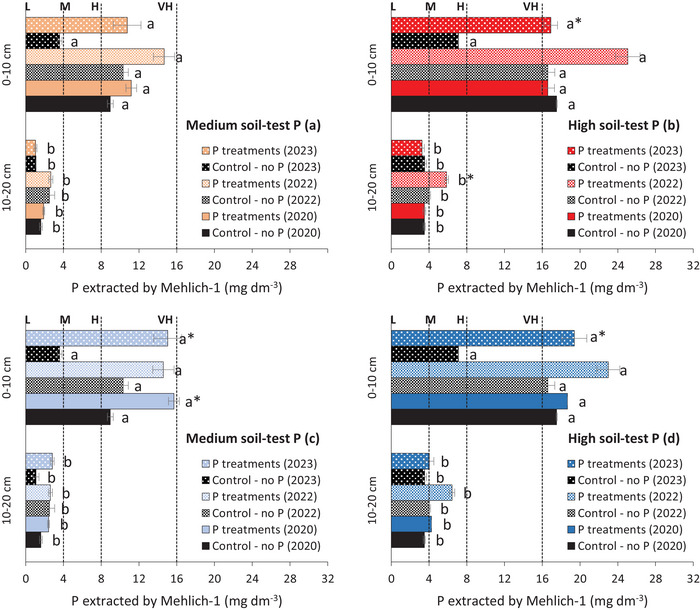
Available P in the soil in the 0–10 and 10–20 cm layers after the first (2020), third (2022) and fourth (2023) year of conducting the experiments under conditions of medium and high soil‐test P: Fertilization time × placement (a and b); Fertilization time × spatial distribution (c and d). Values showed on figures a and b are the average of three fertilization time and two placements, in addition to the control (no P) (*n* = 21 in soil with Medium soil‐test P, *n* = 28 in soil with high soil‐test P); Values showed on figures c and d are the average of two fertilization times and two spatial distribution, in addition to the control (no P) (*n* = 15 in soil with Medium soil‐test P, *n* = 20 in soil with high soil‐test P). Means between layers followed by the same letter are not statistically different according to the Tukey test (*p* < 0.05). Means in the same layer followed by (*) are statistically different from the control, compared by the Wilcoxon Signed–Rank test (*p* < 0.05). L, low; M, medium; H, high; and VH, very high.

The available P was influenced by the soil layer in all evaluations, consistently being higher in the 0–10 cm layer than in the 10–20 cm layer (Table 4). In treatments with phosphate fertilization in the experiment with medium soil‐test P, the available P in the 0–10 cm layer increased over time, reaching approximately twice the CSTV (8 mg dm^−3^ by Mehlich‐1) in the third and fourth years of the experiment (14.7 and 10.8 mg dm^3^, respectively) (Figure 3a). However, in the 10–20 cm layer, the available P was much lower, ranging from 13% to 33% of CSTV (1.0 to 2.7 mg dm^3^, respectively). In the treatments with phosphate fertilization in the experiment with high soil‐test P, the available P in the 0–10 cm layer was 2.9 and 2.4 times higher than the CSTV (23.0 and 19.4 mg dm^3^, respectively) in 2022 and 2023 (Figure 3b). In the 10–20 cm layer, it ranged from 4.0 to 5.9 mg dm^3^, classified as medium and, therefore, below the CSTV.

**TABLE 4:** Summary of statistical results of two comparisons on system fertilization and their interactions on P availability in the soil and crop yield in field trials in soils with soil‐test P medium and high.

Variables	Fertilization time (FT)	Placement (P)	Depth (D)	FT × P	FT × D	P × D	FT × P × D
	ANOVA 1						
**Medium soil‐test P**							
Soil available P in 2020	0.092	0.100	**<0.001**	0.075	0.834	0.082	0.356
Soil available P in 2022	0.156	0.595	**<0.001**	0.943	0.251	0.762	0.880
Soil available P in 2023	0.478	0.071	**<0.001**	**<0.001**	0.649	0.110	0.835
Black oat DM 2019	0.420	**0.004**	–	0.552	–	–	–
Soybean yield 2019/2020	0.744	0.157	–	0.326	–	–	–
Barley yield 2020	0.647	0.064	–	0.391	–	–	–
Soybean yield 2020/2021	0.678	0.625	–	0.357	–	–	–
Soybean yield 2021/2022	**0.019**	0.648	–	**0.016**	–	–	–
Corn yield 2022/2023	0.473	0.608	–	0.415	–	–	–
**High soil‐test P**							
Soil available P in 2020	0.650	0.365	**<0.001**	0.238	0.172	0.675	0.859
Soil available P in 2022	**0.039**	0.792	**<0.001**	**0.030**	**0.037**	0.685	0.188
Soil available P in 2023	**<0.001**	0.849	**<0.001**	0.984	**<0.001**	0.333	0.142
Black oat DM 2019	0.403	0.975	–	0.121	–	–	–
Soybean yield 2019/2020	0.761	0.449	–	0.735	–	–	–
Corn yield 2020/2021	0.447	0.555	–	0.452	–	–	–
Barley yield 2021	0.845	0.320	–	0.267	–	–	–
Soybean yield 2021/2022	0.766	0.128	–	0.682	–	–	–
Soybean yield 2022/2023	0.248	0.067	–	0.549	–	–	–

Variables	Fertilization time (FT)	Spatial distribution (SD)	Depth (D)	FT × SD	FT × D	SD × D	FT × SD × D
	ANOVA 2						
**Medium soil‐test P**							
Soil available P in 2020	**0.004**	0.054	**<0.001**	0.079	0.304	0.238	0.212
Soil available P in 2022	0.161	0.422	**<0.001**	0.207	0.137	0.344	0.607
Soil available P in 2023	0.986	0.295	**<0.001**	0.470	0.158	**0.011**	0.416
Black oat DM 2019	0.993	**0.027**	–	0.941	–	–	–
Soybean yield 2019/2020	0.903	0.224	–	0.564	–	–	–
Barley yield 2019	0.354	0.971	–	0.235	–	–	–
Soybean yield 2020/2021	0.797	0.708	–	0.990	–	–	–
Soybean yield 2021/2022	**0.002**	**0.010**	–	**0.003**	–	–	–
Corn yield 2022/2023	0.394	0.103	–	0.732	–	–	–
**High soil‐test P**							
Soil available P in 2020	0.106	0.220	**<0.001**	0.732	0.951	0.667	0.622
Soil available P in 2022	0.277	0.479	**<0.001**	**0.048**	0.247	0.634	0.402
Soil available P in 2023	0.303	0.137	**<0.001**	0.904	0.839	0.441	0.278
Black oat DM 2019	0.861	0.896	–	0.858	–	–	–
Soybean yield 2019/2020	0.786	0.157	–	0.625	–	–	–
Corn yield 2020/2021	0.997	0.981	–	0.605	–	–	–
Barley yield 2021	0.117	0.196	–	0.942	–	–	–
Soybean yield 2021/2022	0.110	**0.031**	–	0.548	–	–	–
Soybean yield 2022/2023	0.832	0.866	–	0.139	–	–	–

*Note*: Analysis of variance (ANOVA) 1: Fertilization time (90, 45, and 0 DBS) and placement (banded P and broadcast P); ANOVA 2: Fertilization time (90 and 45 DBS) and spatial distribution (17 × 5 and 40 × 10 cm). The bold values indicate statistical significance at *p* < 0.05.

Abbreviations: DBS, days before sowing winter crop; DM: Dry matter; ns, not significant.

**FIGURE 4:**
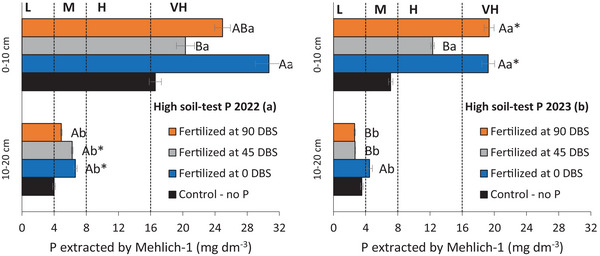
Available P in the soil in the 0–10 and 10–20 cm layers in different fertilization times (90, 45, and 0 days before sowing winter crop—DBS) under high soil‐test P soil on the third (2022) (a) and fourth year of evaluation (2023) (b). Means between layers followed by the same letter are not statistically different according to the Tukey test (*p* < 0.05). Means in the same layer followed by (*) are statistically different from the control, compared by the Wilcoxon Signed–Rank test (*p* < 0.05). L, low; M, medium; H, high; and VH, very high.

Fertilization timing at the high soil‐test P site affected available P in soil layers during years 3 and 4 (Table 4; Figure 4). Despite the soil P gradient, fertilization at 90 or 0 DBS similarly increased available P in the 0–10 cm layer after 3 (Figure 4a) and 4 years (Figure 4b). In the final evaluation, despite an overall decline in available P in the 10–20 cm layer, the 0 DBS application presented values higher compared to the 45 and 90 DBS (Figure 4b).

#### 3.1.2 Fertilization time versus spatial distribution

In both sites (medium and high soil‐test P), fertilization had the greatest effect in the surface 0–10 cm layer compared to 10–20 cm (Table 4). In medium soil‐test P, available P in the 0–10 cm layer ranged from 14.6 to 15.7 mg dm^−3^, while in 10–20 cm it ranged from 2.4 to 2.8 mg dm^−3^ (30%–35% of CSTV) over 3 years (Table 4; Figure 3c). In high soil‐test P, available P in the 0–10 cm layer was 2.3 to 2.9 times the CSTV, while in 10–20 cm it ranged from 50% to 80% of CSTV (4.0–6.4 mg dm^−3^) during monitoring (Figure 3d).

**FIGURE 5:**
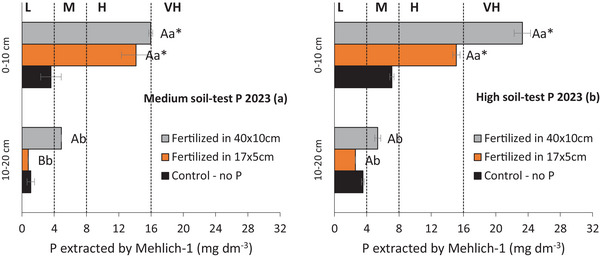
Available P on soil in 0–10 and 10–20 cm layers on different spatial distribution (17 × 5 and 40 × 10 cm) (2023) under conditions of medium (a) and high soil‐test P (b). Means between layers followed by the same letter are not statistically different according to the Tukey test (*p* < 0.05). Capital letters compare spatial distribution (17 × 5 and 40 × 10 cm) at same soil layer; Lowers letters compare soil layers. Means in the same layer followed by (*) are statistically different from the control, compared by the Wilcoxon Signed–Rank test (*p* < 0.05). L, low; M, medium; H, high; and VH, very high.

After 4 years of phosphate fertilization at the medium soil‐test P site, available P in 0–10 cm layer was similar between spatial distributions, ranging from 14.1 to 16.0 mg dm^−3^ (Table 4; Figure 5a). However, fertilization at 40 × 10 cm increased available P in the 10–20 cm layer to 4.9 mg dm^−3^ (61% of CSTV), compared to 0.8 mg dm^−3^ with 17 × 5 cm spacing. In the high soil‐test P site, after 4 years of phosphate fertilization, available P in the 0–10 cm layer increased by 1.9–2.9 times above the CSTV (Table 4; Figure 5b). In the 10–20 cm layer, available P ranged from 32 to 68% of CSTV (2.6–5.4 mg dm^−3^) (Figure 5b).

### 3.2 Effects on grain yield and P efficiency use

#### 3.2.1 Fertilization time versus placement method

**FIGURE 6:**
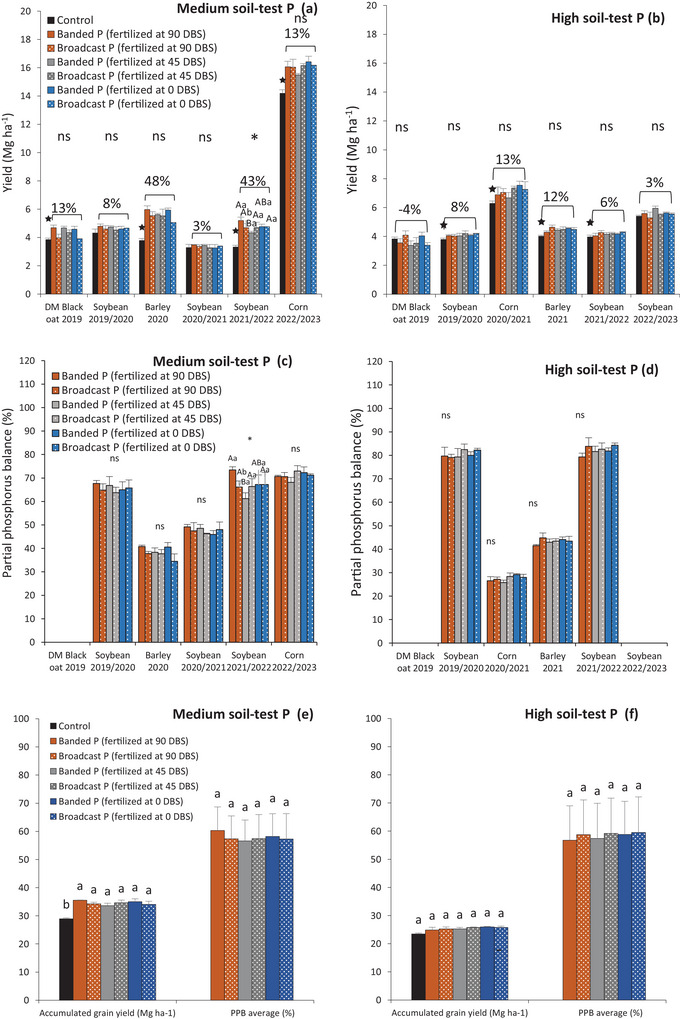
Crop yield over the 4 years of experiment (2019–2023) under different phosphate fertilizer management (fertilization time × placement): in conditions of medium (a) and high soil‐test P (b). Partial phosphorus balance ([P exported by grains and P applied ratio] × 100) of the crops grown in medium (c) and high soil‐test P (d) in a Brazilian subtropical Oxisol. Accumulated yield grain (Mg ha^−1^) and Partial phosphorus balance (%) average of the crops grown in medium (e) and high soil‐test P (f) in a Brazilian subtropical Oxisol. Means followed by the same letter are not statistically different according to the Tukey test (*p* < 0.05). ns: not significant. Capital letters compare fertilization time (90, 45, and 0 days before sowing winter crop) within each placement method (band and broadcast); Lowercase letters compare the placement method (band and broadcast) in each fertilization time (90, 45, and 0 days before sowing winter crop—DBS). *p < 0.05; ns: Not significant. ★: Control average (without P) statistically different from the average of treatments that received P application by the Wilcoxon Signed–Rank test (*p* < 0.05). Percentage values show the change in crop yields that received P fertilizer compared to control treatment.

Only soybean yield and PPB in the medium soil‐test P site during 2021/2022 were affected by the interaction of fertilization time and P placement (Table 4; Figure 6a,c). Phosphorus applied 90 DBS in the seeding furrow increased soybean yield by 11% (5.2 Mg ha^−1^) compared to broadcast application (4.7 Mg ha^−1^). Among different application timings in the seeding furrow, yields were 5.2, 4.3, and 4.8 Mg ha^−1^ for fertilization at 90, 45, and 0 DBS, respectively (Figure 6a). Similarly, for PPB, fertilization performed 90 DBS in the seeding furrow increased PPB by up to 7% compared to other application timings during the 2021/2022 soybean season (Figure 6c).

Considering the total of 12 evaluated crops, with four growing seasons in each soil‐test P condition, medium (Figure 6a) and high (Figure 6b), significant yield differences were observed between treatments with and without P application. However, yield increases were more pronounced in the medium soil‐test P site (29% ± 11%) compared to the high soil‐test P site (4% ± 4%) when treatments with P were compared to the control.

#### 3.2.2 Fertilization time versus spatial distribution

**FIGURE 7:**
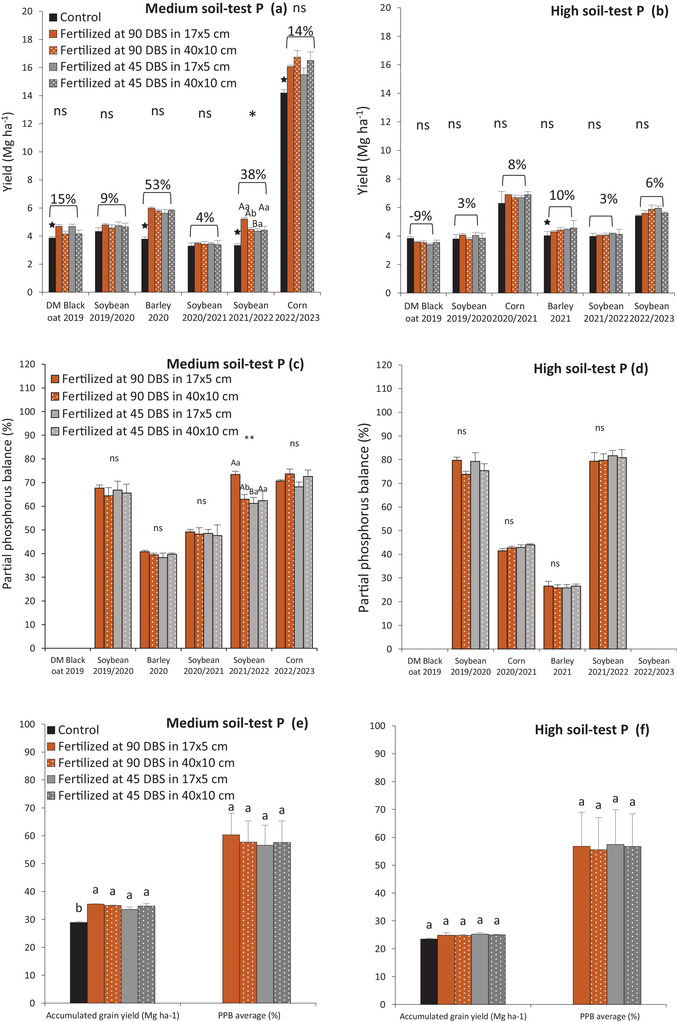
Crop yield over the 4 years of experiment (2019–2023) under different phosphate fertilizer management (fertilization time × spatial distribution): in conditions of medium (a) and high soil‐test P (b). Partial phosphorus balance ([P exported by grains and P applied ratio] × 100) of the crops grown in medium (c) and high soil‐test P (d) in a Brazilian subtropical Oxisol. Accumulated yield grain (Mg ha^−1^) and Partial phosphorus balance (%) average of the crops grown in medium (e) and high soil‐test P (f) in a Brazilian subtropical Oxisol. Means followed by the same letter are not statistically different according to the Tukey test (*p* < 0.05). ns: not significant. Capital letters compare fertilization time (90 and 45 days before sowing winter crop—DBS) within each spatial distribution (40 × 10 and 17 × 5 cm); Lowercase letters compare the spatial distribution (40 × 10 and 17 × 5 cm) in each fertilization time (90 and 45 days before sowing winter crop—DBS). *p < 0.05; **p < 0.01; ns: Not significant. ★: Control average (without P) statistically different from the average of treatments that received P application by the Wilcoxon Signed–Rank test (*p* < 0.05). Percentage values show the change in crop yields that received P fertilizer compared to control treatment.

In medium soil‐test P, fertilization time and spatial distribution affected soybean yield only in 2021/2022 season (Figure 7a). At 17 × 5 cm spacing, soybean yield increased by 0.9 Mg ha^−1^ with fertilization at 90 DBS. At the same timing, soybean yield increased 0.7 Mg ha^−1^ more with 17 × 5 cm spacing than with 40 × 10 cm. Regarding PPB, there was up to a 10% increase in P export when fertilized 90 DBS with a 17 × 5 cm compared to other treatments (Figure 7c).

Across 12 seasons and both soil‐test P levels, five showed significant yield differences between treatments with and without P application: four in medium P (Figure 7a) and one in high P site (Figure 7b). Additionally, yield increases were more pronounced in the medium soil‐test P site (30 ± 11%) compared to the high soil‐test P site (10% ± 4%) when P treatments were compared to the control.

## 4 DISCUSSION

### 4.1 Evolution of the available P in‐depth and its effect on crop yields

The highest concentration of available P was observed in the topsoil during all evaluations (Table 4; Figure 3), which can be attributed to the combined effects of low P mobility in the soil, deposition of plant residues, and the absence of soil tillage under no‐till management (Oliveira et al., 2022; Tiecher et al., 2023). However, the effects of fertilization time (Figure 4b) and spatial distribution (Figure 5b) on the available P in the 10–20 cm soil layer became evident only after 4 years of phosphate fertilization. In soils with long‐term P fertilization, there is a reduction in P adsorption capacity due to the saturation of binding sites, which reduces the soil's buffering capacity and enhances the effectiveness of phosphate fertilization (Barrow & Debnath, 2014).

Phosphate fertilization carried out at winter crop sowing also contributed to increased P levels in the 10–20 cm soil layer over time (Figure 4b). Given the rapid reaction of phosphate ions with soil functional groups, P application at sowing is a common strategy to enhance availability during early crop development. Although it did not increase yield (Figure 6b), the subsurface P rise is agronomically beneficial, supporting root growth and resilience under water stress (Bellinaso et al., 2021). In these trials, the 0–10 cm layer already had sufficient P to support crop performance. Thus, with high surface P, applying fertilizer 90 DBS did not reduce overall soil P availability (Figure 4b).

The highest P increase in the 10–20 cm layer occurred with the 40 × 10 cm distribution (Figure 5a), likely due to lower row density (250 rows ha^−1^), concentrating more P per band than the 17 × 5 cm layout (588 rows ha^−1^) (Fernández; Schaefer, 2012; Oliveira et al., 2022). Chen et al. (2022) showed that subsurface P application mitigates low P in deeper layers. However, in these clayey, highly adsorptive soils, its effects were gradual and only appeared after 4 years of fertilization (Figure 5a).

The greatest yield increase between P‐treated and control treatments in the medium soil‐test P condition (Figures 6a and 7a) demonstrates a greater response to phosphate fertilization in soils with P availability below the CSTV. Furthermore, these results confirm the regional CSTV's adequacy (Fontoura et al., 2015). Interestingly, in the high soil‐test P site, two barley seasons showed a significant increase in yield compared to the control (Figures 6b and 7b), highlighting that barley, with its low P‐use efficiency, is highly responsive to fertilization (Vieira et al., 2015; Yu et al., 2021). This highlights the importance of replacement P rate even in soils with high P availability due to the high P export in intensive grain production systems.

### 4.2 Effects on grain yield and phosphorus use efficiency

Banded P proved effective in increasing soybean yield (Figure 6a), although it typically resembles broadcast fertilization in terms of grain productivity (Freiling et al., 2022). However, P application in the sowing row was particularly effective in increasing P levels in deeper soil layers in soils with a long history of fertilization (Oliveira et al., 2022).

System fertilization, which favors nutrient cycling and soil quality (Camargo et al., 2024; Simões et al., 2023), was also effective in this study. In soils with medium soil‐test P, applying P at 90 DBS resulted in increased soybean yield in the subsequent crop (Figures 6a and 7a). However, this occurred in only one of six seasons evaluated in the medium soil‐test P site and not in any seasons in the high soil‐test P site. This result can be attributed to early fertilization when summer crop residues remained on the field, with an average C:N ratio that stimulated soil microbial activity. This suggests that P applied at this time may have favored microbial P cycling, emphasizing the role of soil microorganisms in P cycling (Martinazzo et al., 2007; Rheinheimer et al., 2008; Tiecher et al., 2012).

At 90 DBS, the greatest increase in yield and P use efficiency in soybean was with the 17 × 5 cm spatial distribution (Figure 7a,c), due to better P homogenization across the total sowing area. The higher number of sowing rows (588 rows ha^−1^) compared to 40 × 10 cm (250 rows ha^−1^) increased the homogenization of the topsoil and favored the P diffusion to the roots. This result was observed in only one of six seasons in the medium P availability site and was not seen in the high soil‐test P site. It was also questioned whether the summer crop would benefit more if the P fertilization in the system fertilization (winter) were applied in the spatial distribution typically used in summer crops (40 × 10 cm). However, the results indicated that applying P closer to the winter crop seed was more beneficial, as it ensured better P utilization, as demonstrated in wheat (Lu et al., 2019), and promotes P cycling for the subsequent crop.

The results suggest that system fertilization can be effective for grain production in both winter and summer crops, provided the available P levels in the 0–10 cm soil layer are above CSTV. Although placement had no consistent effect, banded P is recommended to reduce surface runoff losses and mitigate eutrophication risks (Dodd & Sharpley, 2016), despite the agronomic focus of this study. Future studies should investigate P distribution in depth with more stratified soil sampling (i.e., layers of 2.5 or 5.0 cm) to avoid underestimating available P using deeper diagnostic layers. Also, testing P placements at greater depths (i.e., 15 or 20 cm) should be considered, as increases in 10–20 cm P after 4 years did not translate into yield gains. The results highlight the need for long‐term trials, as no significant water deficits occurred during the 4 years. This should be considered in future studies, especially under climate change, where the distribution of immobile nutrients like P directly affects nutrient use efficiency (Bellinaso et al., 2021).

## 5 CONCLUSIONS

Over 4 years, system fertilization in medium‐ and high‐P subtropical Oxisols under no‐till had minimal effects from fertilization time, placement, or spatial distribution on available P, yield, and P use efficiency.

The predominance of the P gradient was observed in all soil evaluations, where the 0–10 cm soil layer was high or very high in P and sufficient to sustain crop yields, given the adequate precipitation for the crops, explaining the low response to the tested factors under subtropical conditions. However, advancing P fertilization by 90 days for winter crop sowing, applying it in the sowing row, may be feasible, provided the available P in the 0–10 cm soil layer is above the CSTV of P.

After 4 years, P availability increased in the 10–20 cm layer under both soil‐test P conditions. Applying P at 10‐cm depth with 40 cm spacing in soils with medium soil‐test P, as well as in high soil‐test P, and fertilizing at winter crop sowing effectively maintained subsurface P levels.

## AUTHOR CONTRIBUTIONS

**Andria Paula Lima**: Conceptualization; data curation; formal analysis; investigation; methodology; validation; visualization; writing—original draft; writing—review and editing. **Sandra M. V. Fontoura**: Conceptualization; funding acquisition; project administration; resources; writing—review and editing. **Dayana Jéssica Eckert**: Data curation; formal analysis; methodology; validation; writing—review and editing. **Amanda Posselt Martins**: Conceptualization; writing—review and editing. **Renan Costa Beber Vieira**: Conceptualization; writing—review and editing. **Cimélio Bayer**: Conceptualization; visualization; writing—review and editing. **Tales Tiecher**: Conceptualization; data curation; funding acquisition; project administration; resources; supervision; validation; visualization; writing—original draft; writing—review and editing.

## CONFLICT OF INTEREST STATEMENT

The authors declare no conflicts of interest.
